# Interpretable deep learning for rotator cuff calcific tendinopathy diagnosis: a multi-center study

**DOI:** 10.1038/s41598-026-51016-w

**Published:** 2026-06-22

**Authors:** Juan Miranda Bautista, Javier Llorente Peris, Fernando Ybáñez Carrillo, Lara Lloret Iglesias, José A. Vega, Pablo Menéndez Fernández-Miranda

**Affiliations:** 1 Radiology Department, Hospital Universitario Rey Juan Carlos, Móstoles, Madrid Spain; 2 Health Research Institute of the Jiménez Díaz Foundation (IIS-FJD), Madrid, Spain; 3 Department of Physical Therapy, Occupational Therapy, Rehabilitation and Physical Medicine, Rey Juan Carlos University, Madrid, Spain; 4 Advanced Computing and E-Science Research Group, Instituto de Física de Cantabria (IFCA), CSIC-UC, Santander, Spain; 5 Department of Morphology and Cell Biology, Universidad de Oviedo, Oviedo, Spain; 6 Facultad de Ciencias de la Salud, Universidad Autónoma de Chile, Santiago, Chile; 7 Escuela Politécnica Superior, Universidad CEU San Pablo, Madrid, Spain

**Keywords:** Computational biology and bioinformatics, Engineering, Health care, Mathematics and computing, Medical research

## Abstract

The reliable deployment of artificial intelligence systems in medical imaging requires high diagnostic performance, robustness and interpretability. In this study, we developed and evaluated two automated frameworks for binary classification of shoulder radiographs (XRs) using deep learning (DL) and hybrid DL–machine learning (ML) approaches. A convolutional neural network (CNN) based on a fine-tuned VGG19 architecture was trained end-to-end on a large, balanced dataset of 4,268 shoulder XRs. In parallel, hybrid models were constructed by extracting deep feature representations from the trained network and combining them with traditional ML classifiers. Model performance was evaluated on independent internal (*n* = 480) and external (*n* = 308) validation sets. Both approaches achieved high discriminative performance. Paired comparison of Receiver Operating Characteristic (ROC) curves using the DeLong test revealed no statistically significant differences between the end-to-end CNN and the hybrid CNN–ML pipeline for either internal validation (AUC 0.956 vs. 0.961) or external generalization (AUC 0.940 vs. 0.942). Model interpretability was assessed using Grad-CAM and SHAP values. Our results suggest that while both frameworks are robust, the end-to-end DL approach offers a more streamlined workflow and more direct visual explainability via saliency maps. These findings support the potential of AI-based tools for shoulder XR analysis; however, prospective real-world validation, assessment under routine prevalence conditions, and direct comparison with human readers are still needed before clinical integration can be established.

## Introduction

Musculoskeletal (MSK) disorders represent a major and growing burden for healthcare systems worldwide, driven by population aging and the increasing prevalence of chronic conditions^1^. In radiology departments, this has translated into a sustained rise in imaging demand, particularly for conventional radiography, which remains the first-line modality for many MSK indications. This increasing workload has not been accompanied by a proportional expansion of human and structural resources, resulting in reporting delays, diagnostic fatigue, and workflow inefficiencies^2^.

Artificial intelligence (AI) has emerged as a promising strategy to address these challenges by supporting image interpretation, improving diagnostic consistency, and optimizing radiological workflows. In recent years, deep learning (DL)–and in particular convolutional neural networks (CNNs)–have demonstrated expert-level performance across a wide range of radiological tasks, including fracture detection, lesion classification, and anatomical segmentation^3–5^. For clinicians, CNNs represent the most widely adopted DL architecture for medical image analysis, owing to their ability to automatically learn hierarchical visual features directly from pixel data^6^. However, successful clinical translation of these systems depends not only on diagnostic performance, but also on robustness, interpretability and reproducibility under real-world conditions.

Within MSK radiology, AI research has predominantly focused on advanced imaging modalities such as magnetic resonance imaging (MRI) and ultrasound (US), especially for the evaluation of rotator cuff tears^7^. In contrast, the application of AI to conventional shoulder radiographs (XRs) has received comparatively limited attention, despite radiography being the most accessible, standardized, and widely used imaging modality in routine clinical practice^8^.

Calcifying tendinopathy of the rotator cuff (CTRC) is a common and potentially disabling condition characterized by calcium hydroxyapatite deposition within rotator cuff tendons^9^. Diagnosis relies primarily on conventional radiography, where calcifications may be subtle, projection-dependent, and prone to interpretative pitfalls, particularly in high-throughput clinical environments^10^. To our knowledge, we are not aware of any previously described AI-based system specifically developed for the automated detection of CTRC using standard shoulder XRs.

Beyond diagnostic accuracy, the choice of modeling strategy represents a key methodological consideration for clinical AI systems. While end-to-end CNNs have shown strong performance in image classification tasks, hybrid approaches that combine deep feature representations with classical machine learning (ML) classifiers are often evaluated as alternative modeling strategies, particularly in scenarios involving limited or heterogeneous data, with potential advantages in terms of computational efficiency, modularity, and adaptability^11,12^.

In this study, we developed and evaluated an automated framework for binary classification of shoulder XRs according to the presence or absence of CTRC. We systematically compared a pure DL approach with hybrid CNN–ML models, assessing diagnostic performance across both internal and independent prospective external validation sets, formally testing differences in AUC between models, and examining model explainability. By doing so, this work aims not only to advance automated analysis of shoulder XRs, but also to provide practical insights into the trade-offs between different modeling strategies for deployment in routine MSK radiology.

## Materials and methods

**Fig. 1:**
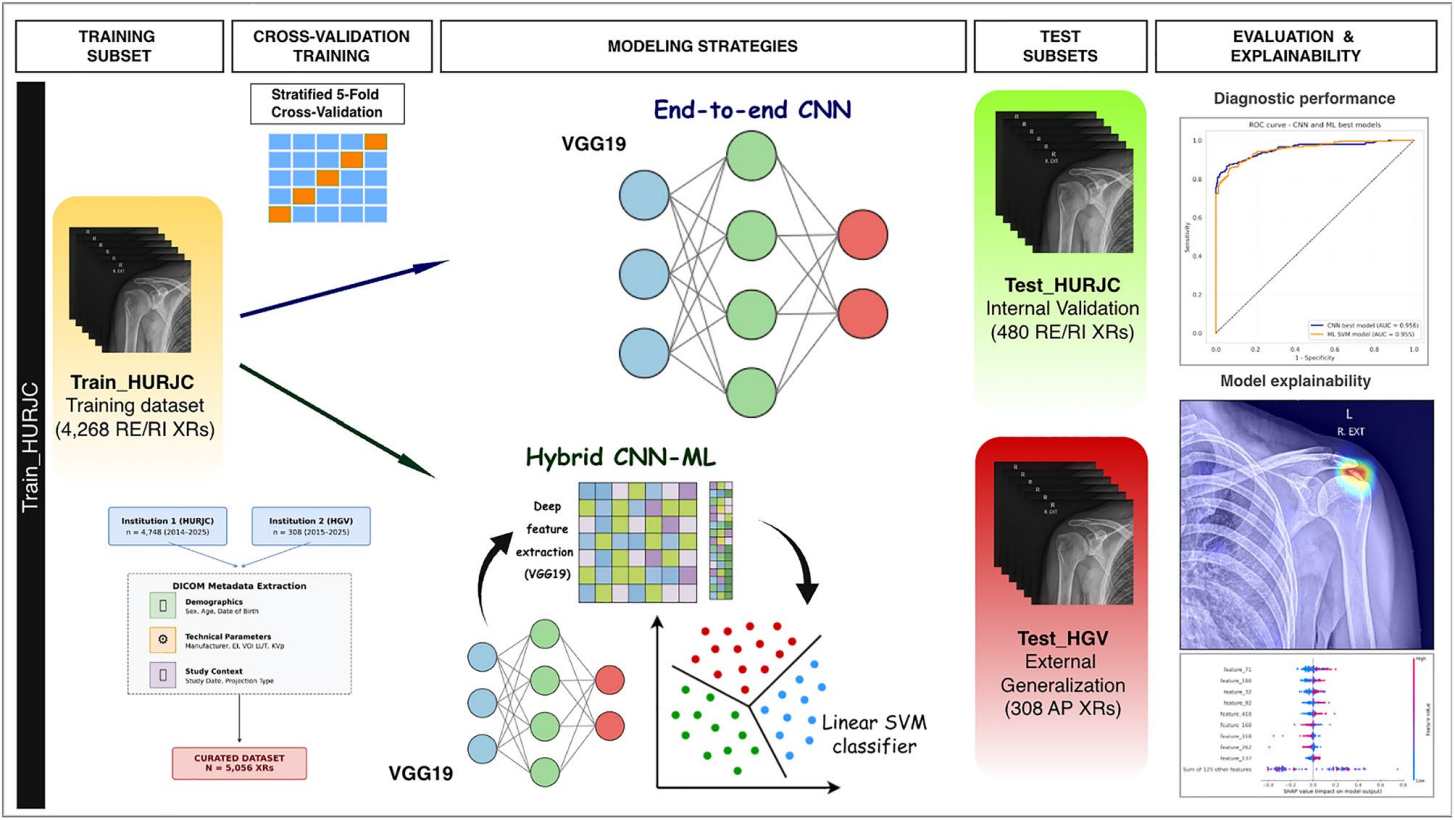
Overview of the experimental workflow.

This retrospective multicenter study involved patients from two institutions located in Spain: Hospital Universitario Rey Juan Carlos (HURJC) and Hospital General de Villalba (HGV). The study presents a unified experimental approach for CTRC detection from routine shoulder XRs, comprising the development of an end-to-end DL model and a complementary hybrid CNN-ML framework designed to balance diagnostic performance, and interpretability. We implemented a unified methodological pipeline consisting of dataset curation and expert labeling, standardized DICOM preprocessing, development of an end-to-end CNN and a hybrid CNN-ML framework under comparable conditions, and evaluation on independent test sets using Receiver Operating Characteristic (ROC) based metrics, paired statistical comparison, and explainability analyses (Fig. 1).

### Ethical approval

This investigation was conducted in accordance with the ethical principles of the Declaration of Helsinki for medical research involving human subjects^13^, as well as relevant national and international regulations for biomedical research. Ethical approval was granted by the Ethics Committee of Fundación de Investigación Sanitaria Fundación Jiménez Díaz, which serves as the governing body responsible for the participating institutions. Given the retrospective nature of the study, the use of anonymized imaging data, and the absence of direct patient interaction, the requirement for informed consent was waived by the Ethics Committee of Fundación de Investigación Sanitaria Fundación Jiménez Díaz.

### Imaging datasets

**Fig. 2:**
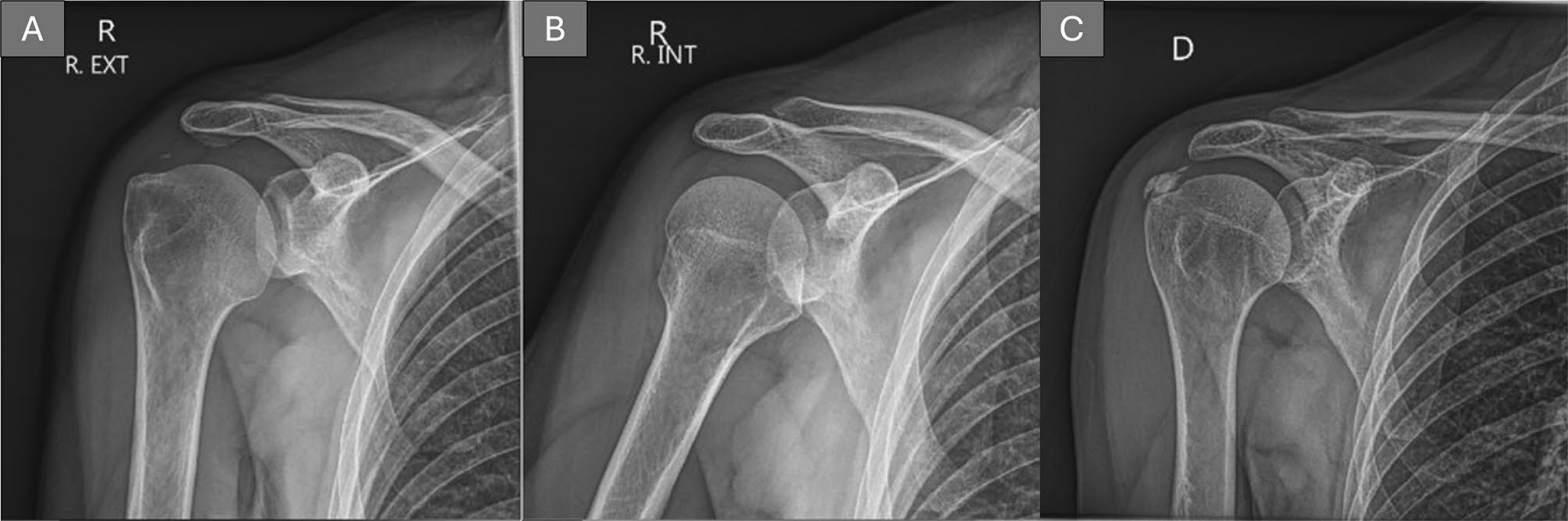
Representative shoulder radiographs showing calcific tendinopathy of the rotator cuff in different projections: (**A**) external rotation, (**B**) internal rotation, and (**C**) anteroposterior views. *CTRC* calcific tendinopathy of the rotator cuff, *ER* external rotation, *IR* internal rotation, *AP* antero-posterior, *XRs* X-rays.

A standardized recruitment process based on predefined inclusion and exclusion criteria (Table 1) yielded a dataset of 5,056 shoulder XRs acquired between 2014 and 2025 across two hospitals: 4,748 from HURJC and 308 from HGV. Images from HURJC included both external (ER) and internal rotation (IR) projections, whereas images from HGV were exclusively antero-posterior (AP) projections, following the distribution reported in Table 2. This design enabled the assessment of both external generalization across institutions and generalization across different radiographic projections. Detailed information regarding laterality, sex, and age is likewise provided. Representative examples of these radiographic views are demonstrated in Fig. 2. All XRs were stored in DICOM format, from which demographic data were extracted (sex, age, date of birth, and study date). To prevent patient-level data leakage and reduce overfitting, only one XR per patient was included.

**Table 1:** Study population criteria and diagnostic class definitions. *XR* radiographs, *ER* external rotation, *IR* internal rotation, *AP* antero-posterior, *CTRC* calcific tendinopathy of the rotator cuff, *MSK* musculoskeletal.

Category	Criteria
Inclusion criteria	– Shoulder XRs (2014–2025) in ER/IR (HURJC) or AP (HGV) projections
	– Age$\ge$18 years and availability of demographics DICOM metadata
	– Diagnosis of CTRC (deposits in supraspinatus, infraspinatus,
	teres minor, or subscapularis) or Control (total absence)
	– Labels validated by consensus of$\ge$2 MSK radiologists
	– Adequate diagnostic image quality
Exclusion criteria	– Prior shoulder surgery or implanted hardware
	– Duplicate examinations from the same patient
	– Non-standard projections or positioning
	– Incomplete/corrupted DICOM files or motion artifacts

**Table 2:** Distribution of the radiographic dataset across training and validation sets. *For HGV, all projections were antero-posterior (AP). *Inst.* Institution, *XR* radiographs, *IR* internal rotation, *ER* external rotation, *M* median, *IQR* interquartile range.

Dataset	Inst.	XR n (%)	Projection n (%)		Laterality n (%)		Sex n (%)		Age (years)		Manufacturer	Purpose
			IR/AP*	ER	Right	Left	Fem.	Male	$\bar{X}$(SD)	M (IQR)		
**Train**_**HURJC**	HURJC	**4268 (100)**	2141 (50.2)	2127 (49.8)	2446 (57.3)	1822 (42.7)	2544 (59.6)	1724 (40.4)	52.1 (13.4)	51.5 (17.8)	Canon Inc.	Training
CTRC		2134 (50.0)	1074 (50.3)	1060 (49.7)	1236 (57.9)	898 (42.1)	1399 (65.6)	735 (34.4)	51.8 (10.5)	50.3 (13.7)		
Control		2134 (50.0)	1067 (50.0)	1067 (50.0)	1210 (56.7)	924 (43.3)	1145 (53.7)	989 (46.3)	52.3 (15.8)	52.7 (21.9)		
**Test**_**HURJC**	HURJC	**480 (100)**	242 (50.4)	238 (49.6)	277 (57.7)	203 (42.3)	322 (67.1)	158 (32.9)	48.2 (11.7)	48.5 (11.4)	Canon Inc.	Internal
CTRC		240 (50.0)	122 (50.8)	118 (49.2)	129 (53.8)	111 (46.2)	174 (72.5)	66 (27.5)	49.4 (8.4)	50.0 (8.3)		validation
Control		240 (50.0)	120 (50.0)	120 (50.0)	148 (61.7)	92 (38.3)	148 (61.7)	92 (38.3)	46.9 (14.3)	47.1 (14.4)		
**Test**_**HGV**	HGV	**308 (100)**	308* (100)	0 (0.0)	164 (53.2)	144 (46.8)	166 (53.9)	142 (46.1)	44.6 (12.2)	46.9 (13.6)	Canon (78.6%)	Generalization
CTRC		154 (50.0)	154* (100)	0 (0.0)	72 (46.8)	82 (53.2)	91 (59.1)	63 (40.9)	49.1 (9.6)	50.0 (10.8)	ATS Srl (21.4%)	
Control		154 (50.0)	154* (100)	0 (0.0)	92 (59.7)	62 (40.3)	75 (48.7)	79 (51.3)	40.0 (13.1)	43.1 (19.8)		

XRs from HURJC were used for model development (*Train*_*HURJC*) and (*Test*_*HURJC*), while HGV XRs (*Test*_*HGV*) were reserved exclusively for external validation to evaluate model generalizability. Specifically, the HURJC dataset comprised a retrospective cohort of 4,268 XRs used for training (*Train*_*HURJC*) and a separate, prospectively collected cohort of 480 XRs for internal validation (*Test*_*HURJC*). In this study, prospectively collected refers to XRs enrolled consecutively at the time of acquisition in routine clinical practice according to the predefined criteria, kept fully held out from any model training or tuning until final evaluation. Likewise, the external generalization set (*Test*_*HGV*) was collected prospectively under the same criteria.

Two MSK radiologists with 3 years of post-fellowship subspecialty practice and 7 and 8 years of total radiology experience, respectively, labeled all eligible images into CTRC and control classes. To ensure the highest reliability of the ground truth, labels were established through a consensus-based workflow: both readers initially evaluated the images independently, followed by a joint review session to resolve any diagnostic discrepancies. XRs showing calcific deposits in one or more rotator cuff tendons, including subscapularis, supraspinatus, infraspinatus, or teres minor, were labeled as CTRC, identifying 2,528 CTRC images across the two hospitals as shown in Table 2. Control cases were randomly selected from remaining eligible XRs at each center to match the number of CTRC images, ensuring balanced datasets (1:1 ratio). Control cases showed no evidence of calcific deposits. This dual-projection, consensus-based assessment by subspecialists supported a robust reference standard, as CTRC typically presents with high radiopacity and clear visual markers on standard XRs.

### Image preprocessing

**Fig. 3:**
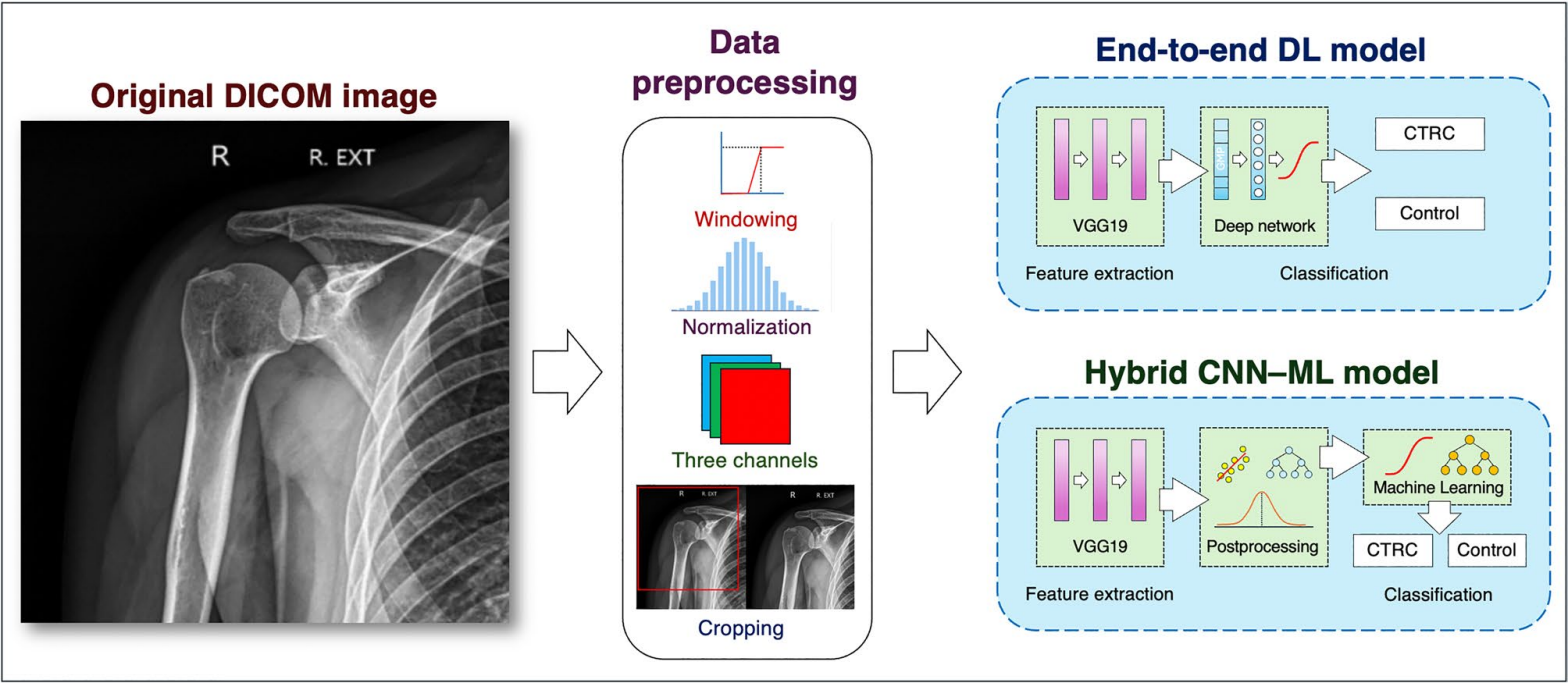
Data preprocessing and modeling pipelines.

All images were processed from the original DICOM files^14^. Radiologist-provided labels and DICOM-derived metadata were integrated using a dual-identifier strategy. Images were irreversibly anonymized using cryptographic hashing^15^. Subsequently, data preprocessing was performed: pixel intensities were inverted when necessary, and windowing was applied. Pixel values were normalized to the [0,1] range. Single-channel grayscale images were converted to three-channel images for compatibility with pretrained CNN architectures. Finally, images were cropped to a square format based on the shortest dimension, applying a laterality-aware horizontal shift to ensure humerus centering, and subsequently resized to $512 \times 512$ pixels. A detailed description of the preprocessing pipeline is provided in Fig. 3

### Training of AI models

#### DL model

An end-to-end DL model based on the VGG19 CNN architecture^16^, pretrained on the ImageNet dataset^17^, was developed for binary classification of shoulder XRs. Transfer learning was applied and all convolutional layers were fine-tuned to adapt pretrained representations to the target task, a strategy widely adopted in medical imaging applications. The convolutional backbone was followed by a global max pooling layer and a final fully connected layer with a single neuron and sigmoid activation to produce class probabilities.

The VGG19 architecture was selected based on its demonstrated success in automated diagnosis across diverse radiographic tasks, including cardiomegaly detection and pneumonia classification^18,19^, as well as its robust performance in broader medical imaging domains such as oncology–specifically for multiclass MRI brain tumor classification and pathology imaging breast cancer diagnosis^20,21^–and dermatology, for the automated detection and classification of skin cancer lesions^22^. This proven effectiveness in extracting hierarchical features, combined with the authors’ prior experience with this model, made VGG19 a suitable choice for the present study.

Model training was performed on the *Train*_*HURJC* set using stratified 5-fold cross-validation (CV) to ensure balanced class representation and robust internal validation. Optimization and training settings were kept identical across folds. The final training configuration was selected after preliminary exploratory experiments. Key architectural and training parameters of the final CNN pipeline, as well as the libraries used and computational resources, are summarized in **Table** S1**of the Supplementary Material**.

#### Hybrid CNN–ML models

A hybrid pipeline was developed by leveraging the feature extractor of a previously trained end-to-end CNN (backbone) and integrating it with classical ML algorithms. Deep feature embeddings were first extracted from the frozen VGG19 backbone^16^ and used to build the feature matrix provided as input to the ML classifiers (Fig. 3). This approach follows a hybrid modeling strategy extensively validated in recent medical image analysis^23–25^.

Prior to training, CNN-extracted features underwent a rigorous post-processing workflow. Highly correlated features were removed using pairwise Pearson correlation (threshold = 0.9) to mitigate collinearity, followed by standardization. Feature importance was estimated using a Random Forest (RF) regressor, selecting the top 139 features for the final classification task. Several algorithms were evaluated, including Logistic Regression (LR), Support Vector Machine (SVM), K-Nearest Neighbors (KNN), Decision Tree (DT), and RF.

These models were selected to represent a range of classical ML classifiers with different decision mechanisms and established use in classification tasks based on deep feature embeddings^25^. Although some of these methods may offer more accessible decision structures than others, the hybrid framework used here does not provide intrinsic human-interpretable features, since classification is performed on high-dimensional deep embeddings. Model optimization was performed via an exhaustive grid search, using stratified 5-fold CV with a fixed random seed ($seed=42$) to ensure reproducibility. The primary optimization metric was the Area Under the ROC Curve (AUC-ROC). Comprehensive hyperparameter configurations and performance metrics for the hybrid models are available in **Table** S2**and Table** S3**of the Supplementary Material**, respectively.

### Performance and explainability evaluation of AI models

The end-to-end DL model and the hybrid CNN–ML frameworks were evaluated on two independent test sets: *Test*_*HURJC* for internal validation and *Test*_*HGV* for external validation to assess model generalizability. Performance was quantified using accuracy, sensitivity, specificity, precision, negative predictive value, F1-score, and the Area Under the Receiver Operating Characteristic curve (AUC), all reported with 95% confidence intervals (CI). Statistical comparisons of the ROC curves were performed using DeLong’s test for correlated samples^26^.

Model explainability was assessed through a multi-level approach: Gradient-weighted Class Activation Mapping (Grad-CAM) was employed to provide visual spatial interpretations of CNN predictions^27^, while SHAP (SHapley Additive exPlanations) values were used to identify feature attribution in the hybrid CNN–ML models^28^. This interpretability framework was further supported by permutation importance analysis to validate the global contribution of deep features, enabling a complementary visual and feature-level understanding of the models’ decision-making processes. Notably, both SHAP and permutation importance analyses were systematically conducted for both the internal validation and external generalization cohorts to ensure the consistency and robustness of the diagnostic drivers across different clinical and technical environments.

## Results

### Diagnostic performance and model comparison

**Fig. 4:**
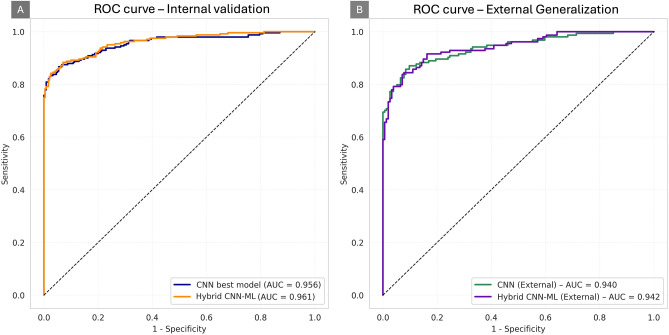
Receiver operating characteristic (ROC) curves of both modeling strategies across (**A**) the internal validation (*Test*_*HURJC*) and (**B**) the external generalization (*Test*_*HGV*) cohorts. *AUC* area under the curve, *CI* confidence interval, *SVM* support vector machine, *CNN* convolutional neural network, *ML* machine learning.

The diagnostic performance of both the end-to-end CNN and the hybrid CNN–ML (SVM) models is shown in Table 3. In the internal validation set (*Test*_*HURJC*, $n = 480$), the end-to-end CNN achieved an AUC of 0.956 [95% CI: 0.937–0.975], while the hybrid model reached an AUC of 0.961 [95% CI: 0.945–0.975]. Statistical comparison using DeLong’s test indicated no significant difference in discriminative capacity between the two approaches ($\Delta$AUC = $-0.0049$, $p = 0.1553$) (Fig. 4).

To evaluate generalizability, models were assessed on the external test set (*Test*_*HGV*, $n = 308$). The CNN and hybrid models yielded AUC values of 0.940 [95% CI: 0.912–0.968] and 0.942 [95% CI: 0.915–0.966], respectively (Fig. 4). Consistent with the substantial overlap of their 95% CIs, the difference remained statistically non-significant ($\Delta$AUC = $-0.0023$, $p = 0.6403$). Detailed results for sensitivity, specificity, precision, and F1-score across both cohorts are also summarized in Table 3.

To ensure the robustness of these findings against variations in class distribution, a Precision-Recall analysis was conducted for both internal and external validation sets (**Supplementary Figure S1**). In the internal validation cohort (*Test*_*HURJC*), the end-to-end CNN and the hybrid model achieved Average Precision values of 0.967 and 0.970, respectively. High discriminative performance was consistently maintained in the external validation set (*Test*_*HGV*), with the CNN yielding an AP of 0.954 and the hybrid model an AP of 0.954.

**Table 3:** Diagnostic performance of both modeling strategies across the internal validation (*Test*_*HURJC*) and external generalization (*Test*_*HGV*) cohorts. *Sens* sensitivity, *Spec* specificity, *Prec* precision (PPV), *Acc* accuracy, *AUC* area under the receiver operating characteristic curve, *CI* confidence interval.

Test Dataset	Sens		Spec		Prec		F1		Acc		AUC (95% CI)	
	DL	Hybrid	DL	Hybrid	DL	Hybrid	DL	Hybrid	DL	Hybrid	DL	Hybrid
Internal Val.	0.850	0.850	0.950	0.958	0.944	0.953	0.895	0.899	0.900	0.904	0.956 (0.937–0.975)	0.961 0.945–0.975)
External Val.	0.857	0.857	0.916	0.890	0.910	0.886	0.883	0.871	0.886	0.873	0.940 (0.912–0.968)	0.942 (0.915–0.966)

### Model explainability

#### Interpretability of the end-to-end DL model

**Fig. 5:**
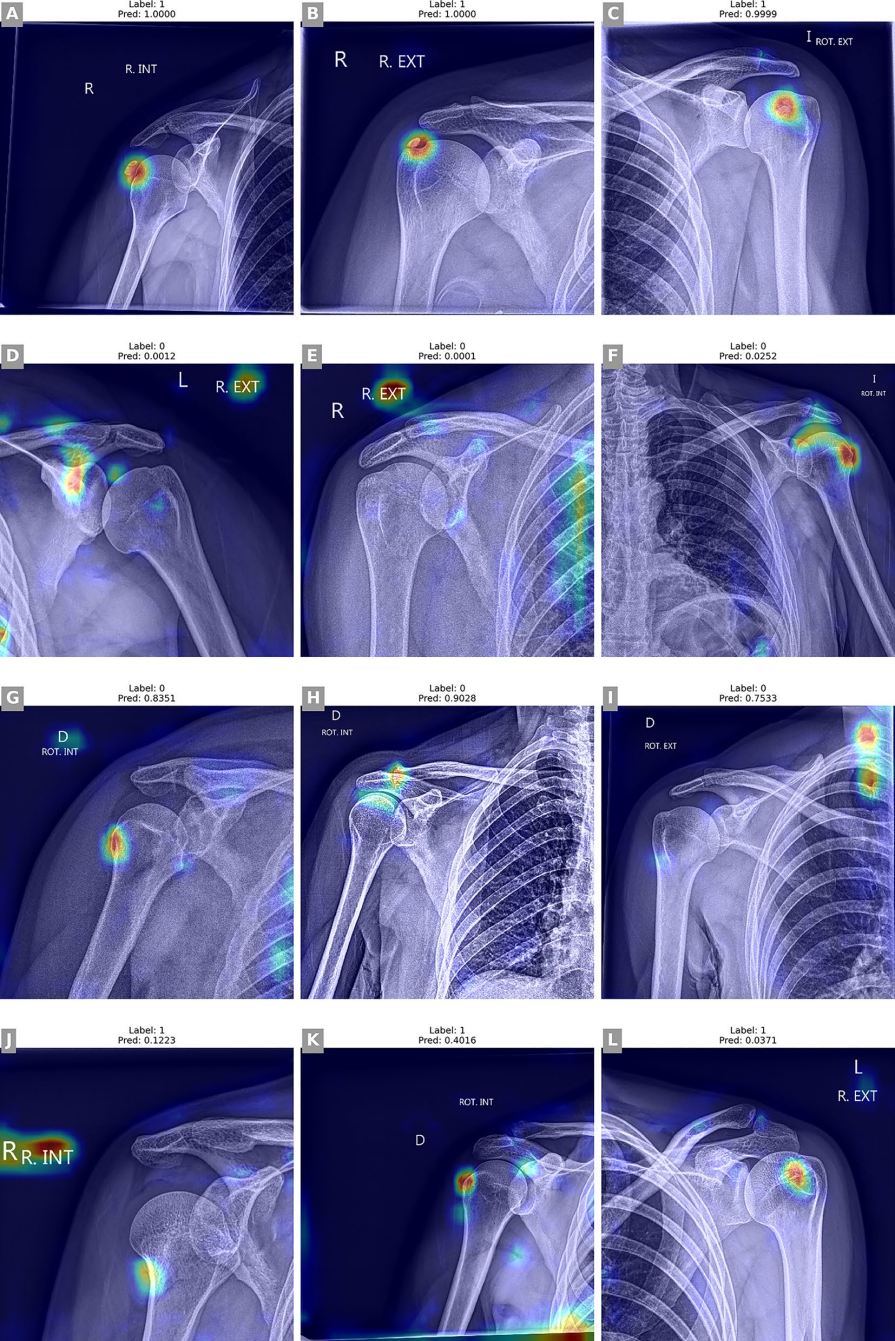
Grad-CAM visualization of CNN attention on representative shoulder XRs from the internal validation (*Test*_*HURJC*) set. The figure includes true positives (**A**–**C**), true negatives (**D**–**F**), false positives (**G**–**I**), and false negatives (**J**–**L**).

**Fig. 6:**
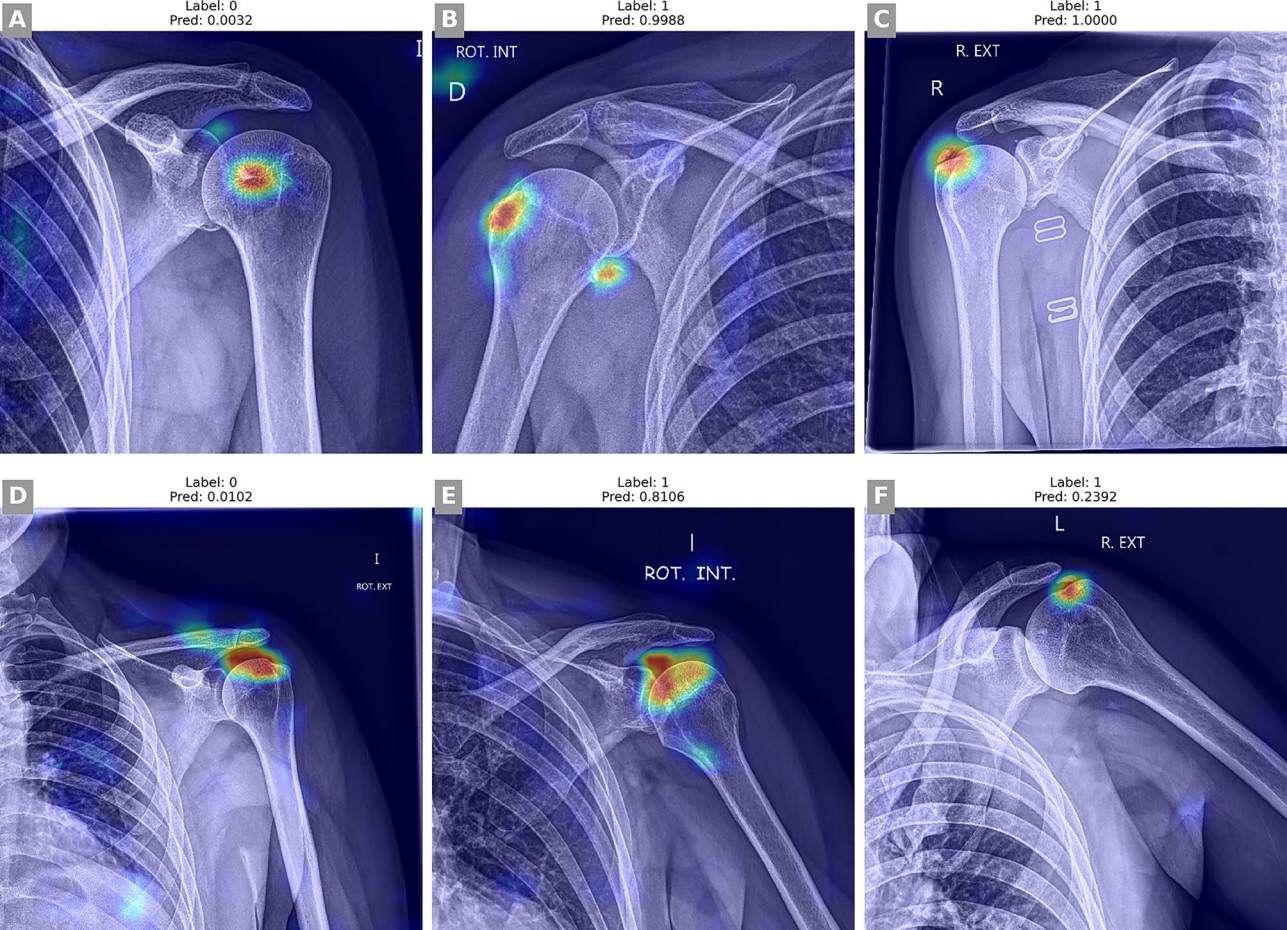
Grad-CAM examples from the internal validation (*Test*_*HURJC*) set illustrating clinically relevant cases and representative diagnostic pitfalls.

Qualitative explainability analysis using Grad-CAM was performed to visualize the regions of interest prioritized by the CNN. Figures 5 and 6 summarize the findings for the internal validation set. Figure 5 shows twelve representative cases: true positives (A–C), true negatives (D–F), false positives (G–I), and false negatives (J–L). Complementarily, Figure 6 highlights six clinically relevant cases, including correct classifications and common radiological pitfalls.

**Fig. 7:**
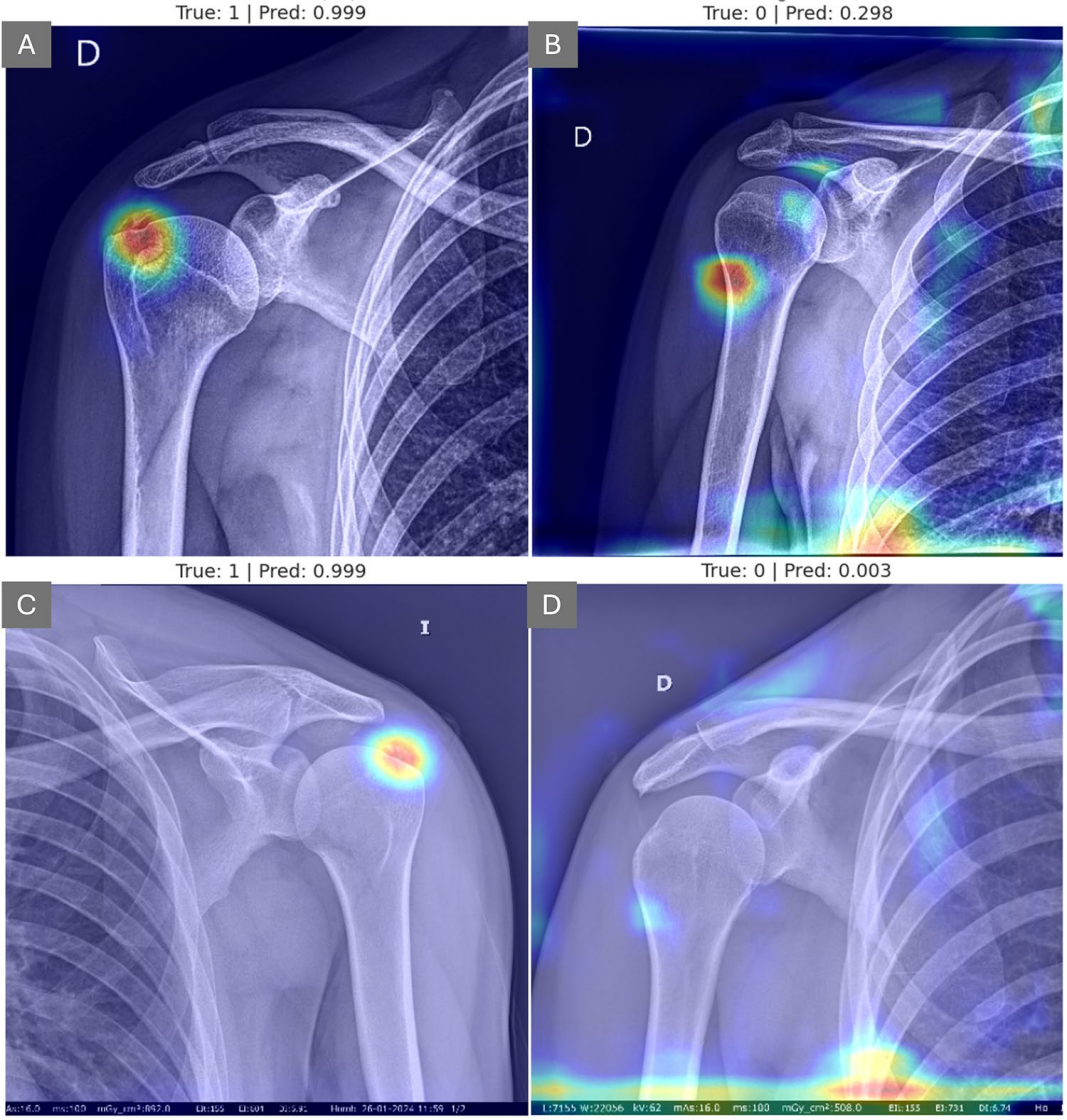
Grad-CAM visualization of CNN attention on representative shoulder XRs from the external generalization (*Test*_*HGV*) set. The figure includes true positives (**A**, **C**) and true negatives (**B**, **D**). Panels A and B correspond to images acquired with Canon hardware, while panels C and D represent ATS Srl hardware.

The Grad-CAM heatmaps from the external generalization set, shown in Fig. 7, illustrate the model’s performance across different imaging devices through four representative examples: true positives (A, C) and true negatives (B, D). Panels A and B correspond to Canon devices, while panels C and D correspond to ATS Srl devices.

#### Explainability of hybrid CNN–ML models

**Fig. 8:**
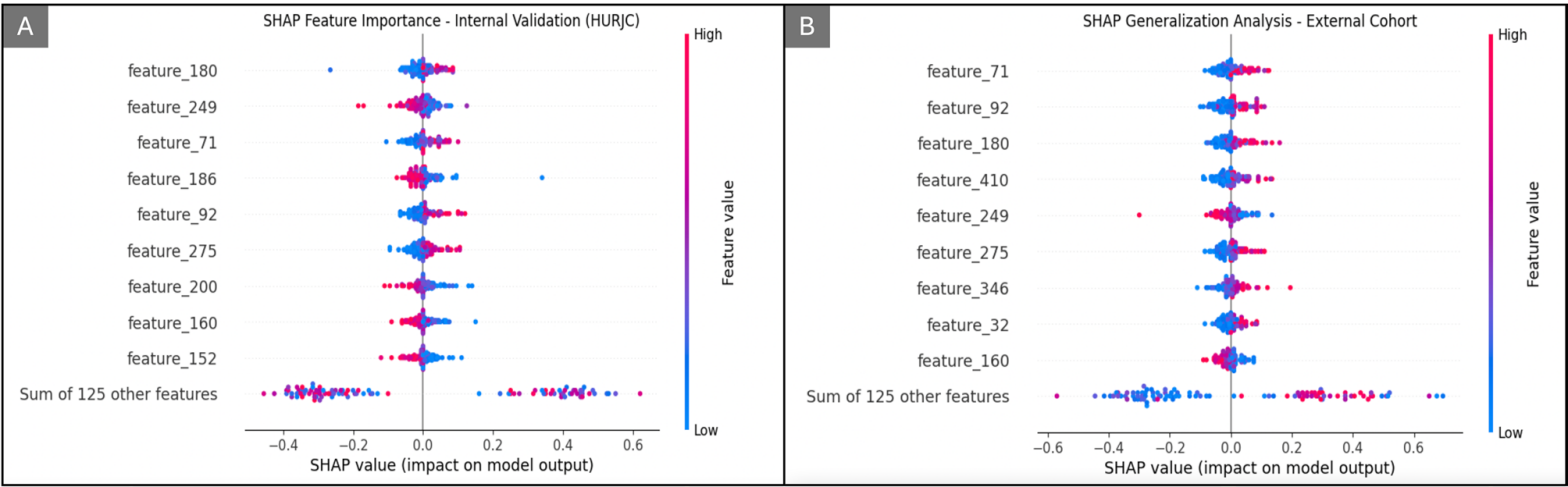
SHAP summary plots for the hybrid CNN–ML model evaluated on the (**A**) internal validation and (**B**) external generalization cohorts. The distribution identifies the top deep features contributing to the diagnostic prediction, demonstrating high consistency in feature attribution (e.g., *feature*_*71*, *feature*_*92*, and *feature*_*180*) across different clinical institutions.

The SHAP analysis identified a sparse and consistent subset of deep features as the primary drivers of model output. Specifically, *feature*_*180*, *feature*_*71*, and *feature*_*92* demonstrated the highest impact on diagnostic prediction in both the internal validation and the external generalization cohorts (Fig. 8).

The results of the global feature relevance identified a stable hierarchy where the top-ranked deep embeddings identified with the RF coincided with the high-impact features observed in the SHAP analysis. In the internal validation set, *feature*_*92*, *feature*_*180*, and *feature*_*71* were identified as the most critical features for maintaining model accuracy (Fig. 8). While the external cohort showed a high reliance on *feature*_*154* and *feature*_*124*, features such as *feature*_*92* and *feature*_*180* maintained their importance across both institutions. The complete permutation importance rankings for both cohorts are provided in **Figure S2 of the Supplementary Material**.

## Discussion

The results of this study suggest that both the end-to-end CNNs and the hybrid CNN–ML approach achieve excellent discriminative capacity in the identification of CTRC, with AUC values of 0.956 [95% CI: 0.937–0.975] and 0.961 [95% CI: 0.945–0.975], respectively. This high diagnostic efficacy is consistent with current literature, as highlighted by He et al.^29^, positioning DL as the current gold standard for anomaly detection in MSK radiography and overcoming the limitations of manual feature engineering. Our findings suggest the maturity of automated systems in identifying complex structural patterns with high clinical reliability.

Specifically, recent evidence demonstrates that CNN-based models can match or surpass the diagnostic accuracy of specialized clinicians across diverse anatomical regions, such as wrist and shoulder, with AUC values consistently exceeding 0.92^30–32^. Beyond traumatic pathology, the robustness of these models has been successfully extended to analyzing degenerative cervical spondylosis and altered spinal cord signal on MRI^33^, and classifying MSK tumors through automated segmentation and predictive classification using DL and radiomics^34^. These advancements consolidate AI as a versatile and multidisciplinary support tool in modern radiology.

A critical observation in our study is the diagnostic parity between both modeling strategies. This empirical finding suggests that the convolutional backbone serves as a highly effective domain transformer, capturing radiological patterns–such as high calcium density relative to soft tissue–so robustly that the resulting hierarchical representations are informative enough on their own, regardless of the downstream classifier. The absence of statistically significant differences and 95% CI overlap between the end-to-end CNN and the hybrid pipeline indicates that the deep features extracted by the VGG19 architecture are sufficiently discriminative.

This finding is further supported by the architecture of our end-to-end model, which utilizes a single output neuron with sigmoid activation following the convolutional backbone. By omitting additional hidden layers in the classification stage, the network is constrained to perform a linear separation in the high-dimensional feature space. The fact that this streamlined design matches the performance of optimized ML algorithms, such as the SVM, suggests that the convolutional architecture successfully projects the XRs into a latent space where healthy and pathological classes are linearly separable. From a computational perspective, this implies that the VGG19 backbone has already resolved the problem’s complexity within its convolutional layers, simplifying the final decision boundary. If the classification task involved complex non-linear dependencies at its final stage, the use of a single neuron would have significantly hindered performance, necessitating a multi-layer perceptron to model such boundaries.

Despite this diagnostic parity, the hybrid CNN–ML pipeline may still offer practical methodological advantages. By decoupling feature extraction from classification, the hybrid strategy enables the use of lightweight ML classifiers (e.g., SVM or RF) that can be retrained or fine-tuned to specific institutional cohorts or new hardware signatures in a fraction of the time and with lower GPU requirements than a full DL recalibration^35^. Ultimately, the hybrid approach serves as a flexible diagnostic tool that leverages the robust feature extraction of the VGG19 backbone while allowing for a more adaptable and resource-efficient integration into diverse hospital information systems.

The choice of VGG19 as a backbone reflects a strategy of architectural parsimony. While newer models like EfficientNet^36^ or Vision Transformers^37^ represent the state-of-the-art in general computer vision, VGG19 remains an established and robust backbone for identifying high-contrast structures in radiography. Recent evidence suggests that VGG-family architectures can offer stable performance in limited-data medical imaging settings, avoiding the over-parameterization and ’data-hunger’ typical of deeper models^18,19,21,22^. Furthermore, its simpler hierarchical structure facilitates the generation of precise, clinical-grade Grad-CAM saliency maps, whereas more complex models often produce noisier or fragmented activations.

Beyond architectural stability, this diagnostic parity also has significant implications for model generalization. As Menéndez Fernández-Miranda et al.^38^ demonstrated, DL models often encounter performance drops when deployed across different centers due to hardware-specific signatures and variations in X-ray device characteristics. This phenomenon is frequently linked to the ”texture bias” inherent in CNNs, as described by Geirhos et al.^39^, where models prioritize local pixel patterns over global anatomical shapes. In our study, the consistent performance across Canon and ATS Srl hardware suggests that the VGG19 backbone has learned to transcend these device-specific textures by prioritizing high-contrast morphological features and anatomical anchors. In the specific task of identifying CTRC, the challenge primarily involves detecting a highly dense, high-contrast structure–calcium–within a defined anatomical region, the rotator cuff. In this context, previously reported biases related to texture recognition may have had limited impact, as the target structure exhibits a substantial contrast and textural difference relative to surrounding tissues, facilitating its discrimination by the model.

Extending this robustness to the spatial domain, a notable finding of our study is the model’s ability to generalize across different radiographic projections. Although our architectures were primarily developed using ER and IR views, they demonstrated effective performance when evaluated on AP projections. This is likely due to the geometric similarity between AP and ER orientations, which facilitated a successful feature transfer of the calcific morphology. However, while this suggests a degree of projection-invariance, such robustness cannot be guaranteed for anatomically distinct views, such as abduction or Scapular Y-projections, which present substantial geometric variances. Given the lack of a universal consensus on shoulder radiographic protocols, this procedural heterogeneity underscores the ongoing challenges of domain shift in real-world DL deployment^38,40^.

This shift from texture to shape is visually confirmed by the model’s transparency, which reveals that its efficacy is rooted in the learning of anatomically coherent representations rather than spurious pixel noise. In pathological cases, Grad-CAM activation maps consistently corresponded to the anatomical location of the rotator cuff. Conversely, in healthy subjects, a systematic exploration pattern was observed across the glenoid, the humeral head, and the subacromial space. This ”selective attention” toward key anatomical landmarks is an emerging strategy in recent medical literature to ensure that AI systems avoid learning shortcuts, reinforcing the model’s robust generalization. Our model focuses on the components of the scapulohumeral joint to evaluate the integrity of the rotator cuff. This structural recognition is critical; as suggested by Raffy et al.^41^, accurate classification in computed tomography and MRI requires the network to fixate on stable structural landmarks–such as the iliac crests or the diaphragm–which is equivalent to our model’s reliance on the glenoid and humerus as reference ”anchors.”

A novel aspect of our findings is the model’s use of these regions to actively rule out pathology. This concept of ”Anatomical Attention Regions”, recently proposed by Nln et al.^42^ for organ segmentation, demonstrates that networks ”recognize” the location of vertebrae before delimiting soft tissues. In our study, the model recognizes the subacromial space as the critical search zone. Translated to our experiment, the network performs a systematic visual triage: if no diagnostic ”footprint of CTRC” is found after evaluating the glenoid and subacromial space, the subject is classified as healthy. This ability to use normal anatomy as ”negative evidence” to rule out pathology confers a diagnostic robustness superior to simple pixel-patch detection.

A finding of particular clinical relevance was the model’s robustness against radiological mimics. Notably, the inclusion of bone islands and metallic artifacts across both the CTRC and control cohorts ensured that these features did not serve as confounding factors. This balanced distribution–carefully maintained throughout the training and validation phases–allowed the system to accurately discriminate findings such as bone islands in the humeral head, which share densitometric similarities with calcifications, as well as extrinsic metallic hardware. The fact that these features did not compromise accuracy suggests that the system relies on subtle textural distinctions and anatomical localization rather than simple pixel intensity. By internalizing these spatial constraints and relying on stable anatomical anchors, the model prevents false positives in regions where calcifications are anatomically improbable.

However, error analysis revealed a sensitivity to technical quality: false positives were frequently associated with excessive noise or poor collimation that included irrelevant structures like the spine. Furthermore, false negatives reflected intrinsic limitations of 2D radiography, such as failing to detect subscapularis calcifications in specific projections or in patients with suboptimal positioning–limitations that are shared by specialists in routine clinical practice.

The hybrid framework provides an intermediate degree of explainability and enhances efficiency by reducing input dimensionality to a subset of 139 attributes via SHAP analysis^28^ without compromising diagnostic accuracy. Beyond this reduction, the consistency in feature attribution between the internal and external cohorts demonstrates the model’s capacity to capture universal signatures of CTRC, ensuring high generalization stability across different institutional contexts. However, a significant interpretability gap remains: these deep features are inherently non-semantic and lack a direct, intuitive correlation for the practitioner. While attributes such as *feature*_*71* or *feature*_*180* are statistically decisive for the model’s internal logic, they offer no immediate physiological insight into the underlying pathophysiology. Nonetheless, this hybrid approach acts as a crucial ”mathematical bridge”; by narrowing the decision-making process to a structured subset of stable descriptors, it moves away from the traditional ”black-box” towards a more transparent, albeit still technical, validation of model consistency.

In contrast, the explainability provided by the end-to-end CNN model offers significantly greater value for radiological practice; unlike the non-spatial quantification of the hybrid pipeline, the saliency maps generated via Grad-CAM provide direct localization that aligns with the radiologist’s visual assessment. This intuitive interpretability acts as a crucial decision-support tool, reinforcing the model’s inference and fostering trust for clinical integration. This preference for visual transparency is consistent with the emerging gold standard in medical AI, where recent end-to-end interpretable frameworks emphasize modeling the radiologist’s intentions through controllable architectures^43–45^. That said, it is important to acknowledge that both Grad-CAM and SHAP provide post-hoc explanations rather than direct causal insights. As such, these visualizations should be interpreted as descriptive maps of model attention–which may occasionally highlight regions correlated with but not causative of the pathology–rather than as absolute indicators of clinical transparency.

Despite the high diagnostic performance achieved, several limitations of this study must be acknowledged. First, the retrospective nature of the dataset necessitates future prospective trials to evaluate the model’s ’real-time’ clinical utility. Second, this study does not include a head-to-head comparison with radiologists. While such a comparison is a logical next step, our primary objective was to establish the technical feasibility of a hybrid AI framework for CTRC detection. In this context, the model is designed as a decision-support tool to optimize workflows, rather than a replacement for radiological expertise. Future research should focus on multi-reader studies to assess the synergistic effect of AI-assisted diagnosis on clinical accuracy and reporting time. Third, the balanced 1:1 sampling strategy excluded common concomitant shoulder pathologies. While this approach was essential to demonstrate the utility of AI in MSK for specifically resolving CTRC–a key step toward optimizing patient workflows–it does not fully reflect the complexity of routine radiology. In clinical practice, overlapping conditions such as advanced osteoarthritis, acute fractures, or degenerative joint disease could introduce ’noise’ into the inference process, potentially affecting false-positive or false-negative rates. Consequently, validating these models on ’unfiltered’ multi-label datasets is a critical requirement for clinical implementation to ensure robustness against real-world disease prevalence. Finally, while the external validation cohort encompasses significant technical variability in device manufacturers and radiographic projections, a specific stratified analysis by subgroup was not performed. Although the sustained high performance across these diverse conditions points toward model robustness, a more granular investigation into hardware-specific influence is warranted. Future multi-center evaluations should incorporate larger, balanced subgroups to formally quantify the impact of specific technical confounders on algorithmic performance.

To bridge the gap between research and clinical practice, we have developed a diagnostic support platform that implements both the end-to-end CNN and hybrid models. This tool–currently in its first iteration–allows for the native processing of DICOM, JPEG, and PNG formats, facilitating its testing with heterogeneous data sources in real-world environments. As a commitment to technological transfer and open science, the application is publicly available as a Hugging Face Space^46^.

## Conclusions

The present study suggests that DL-based approaches, including both end-to-end and hybrid CNN–ML architectures, may support the automation of selected MSK XR tasks and could assist radiologists in controlled settings. Experimental results showed that both strategies achieved high diagnostic accuracy for identifying CTRC across internal and external cohorts, with the end-to-end model showing a slight overall advantage. Performance also remained stable across different imaging projections and an independent hospital cohort, although these findings should be interpreted within the limitations of the present experimental design. By leveraging anatomical consistency and focusing on salient morphological features, the models not only replicate expert radiological reasoning but also provide interpretable outputs that foster clinician trust. Overall, these results support the potential of AI-based tools for shoulder XR assessment, paving the way for broader automation and enhanced efficiency in clinical practice.

## Supplementary Information

Supplementary Information.

## Data Availability

Data from this study were anonymized and can be made available upon reasonable request by contacting the first author at juan.bautista@urjc.es.

## Code availability

Source code is publicly available on GitHub: https://github.com/mirandabautistajuan-lang/shoulder-xray-cnn-hybrid. The imaging data used in this study are not hosted in the repository due to privacy and ethical considerations, in compliance with data protection regulations. However, these data can be provided upon reasonable request to the corresponding author, subject to institutional approvals and formal data-sharing agreements.
