# Supplementary material for: Interpretable deep learning for rotator cuff calcific tendinopathy diagnosis: a multi-center study
Source: Sci Rep. 2026 Jun 22;16:19375. doi: 10.1038/s41598-026-51016-w (PMC13287596; doi:10.1038/s41598-026-51016-w)
Supplement: Supplementary file 1 — Supplementary Information. [file 41598_2026_51016_MOESM1_ESM.pdf]

Supplementary Material

Miranda Bautista et al.

Component	Parameter	Final configuration
**Architecture**	Backbone	VGG19 pretrained on ImageNet; all convolutional layers fine-tuned.
	Input	$512 \times 512 \times 3$ (grayscale XRs converted to 3-channel RGB).
	Pooling	Global max pooling.
	Classifier head	Dense layer (1 unit) with sigmoid activation.
	Output	Binary probability (CTRC vs. control).
**Training**	Optimizer	Stochastic gradient descent (SGD).
	Learning rate	0.005.
	Loss function	Binary cross-entropy.
	Batch size	32.
	Max epochs	50.
	Early stopping	Monitored on validation loss; patience = 15 epochs.
**Validation**	Strategy	Stratified 5-fold cross-validation (4 folds train, 1 fold validation).
	Class balance	Preserved within each fold (stratified splits).
**Data loading**	Data format	Preprocessed XRs stored as NumPy arrays.
	Batch loading	Custom Keras Sequence generator (disk-to-RAM mini-batches).
	Reproducibility	Fixed random seed for shuffling (seed = 42).

Table S1. Configuration of the end-to-end CNN training pipeline. *CNN* convolutional neural network, *VGG19* Visual Geometry Group 19-layer network, *XR* X-ray, *RGB* red green blue, *CTRC* calcific tendinopathy of the rotator cuff, *SGD* stochastic gradient descent, *RAM* random access memory.

Component	Parameter	Methodology / Configuration
**Optimization**	Strategy	Exhaustive grid search (`GridSearchCV`).
	Primary Metric	Area under the ROC curve (AUC-ROC).
	Validation	Stratified 5-fold cross-validation (`seed=42`).
**Model Grids**	Logistic Regression	C: [0.001–100]; Penalty: [ℓ_1, ℓ_2]; Solvers: [liblinear, saga].
	K-Nearest Neighbors	k: [1–20]; Weights: [uniform, distance]; p: [1, 2].
	Support Vector Machine	C: [0.01–100]; Kernels: [linear, rbf, sigmoid, poly]; γ: [scale, auto].
	Decision Tree	Depth: [None, 5–30]; Split/Leaf: [2, 1, 4, 6]; Criterion: [gini, entropy].
	Random Forest	Trees: [50, 100, 200]; Depth: [None, 10–30]; Bootstrap: [T, F].
**Implementation**	Feature Input	Deep features extracted from VGG19 (frozen backbone).
	Software	Scikit-learn framework via `Pipeline` architecture.
	Hardware	Parallelized execution (`n_jobs=-1`).
**Reproducibility**	Seed	Fixed random seed (42) for all stratified splits.
	Persistence	Best estimators exported via `joblib` (.pkl format).

Table S2. Experimental setup for the hybrid CNN–ML framework.

Model	Sens.	Spec.	Prec.	F1	Acc.	NPV	AUC (95% CI)	Time (s)
Logistic Regression	0.892	0.883	0.884	0.888	0.887	0.891	0.961	31.93
K-Nearest Neighbors	0.833	0.942	0.935	0.881	0.887	0.850	0.955	5.55
**Support Vector Machine**	**0.892**	**0.879**	**0.881**	**0.886**	**0.885**	**0.890**	**0.961**	**458.91**
Decision Tree	0.77	0.91	0.90	0.83	0.84	0.80	0.893	25.08
Random Forest	0.88	0.96	0.95	0.91	0.92	0.88	0.976	4860.29

Table S3. Performance comparison of hybrid models during the training and cross-validation phase. Metrics correspond to classifiers trained on deep feature embeddings, with the SVM (highlighted) selected as the optimal model. *Sens.* sensitivity, *Spec.* specificity, *Prec.* precision, *Acc.* accuracy, *NPV* negative predictive value, *AUC* area under the ROC curve.

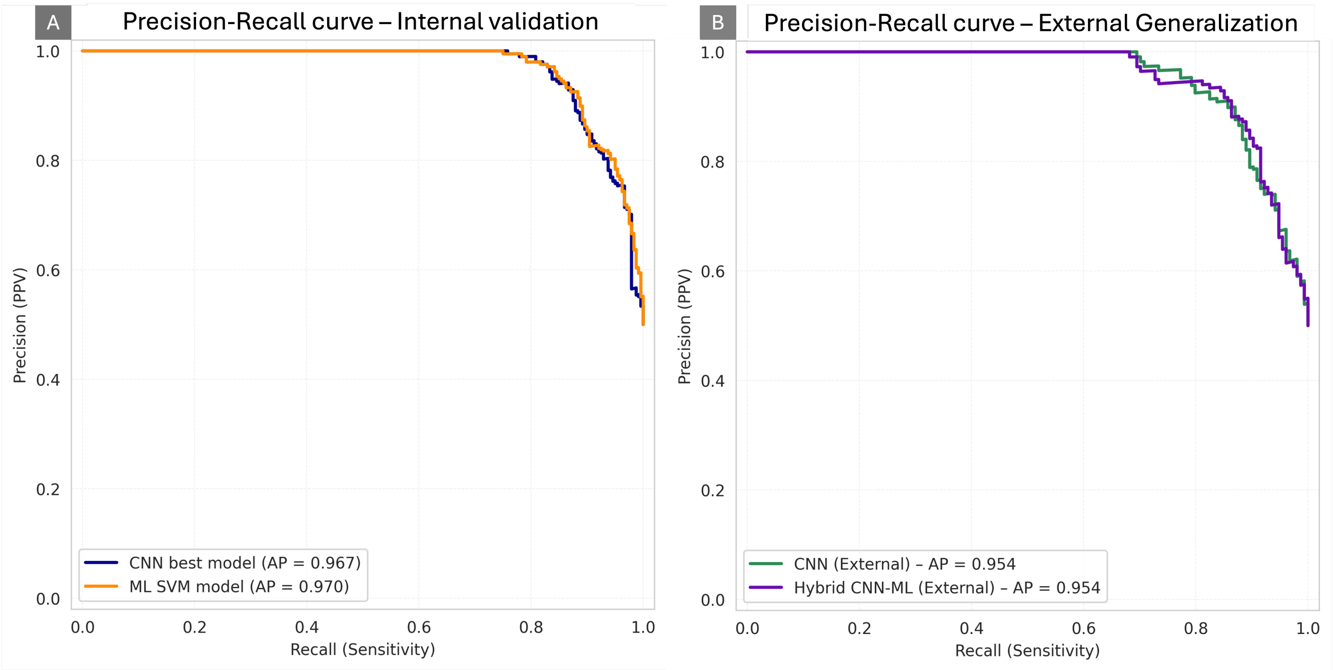

Figure S1. Precision-Recall curves of both modeling strategies across (A) the internal validation (*Test_HURJC*) and (B) the external generalization (*Test_HGV*) cohorts. *AP* average precision, *SVM* support vector machine, *CNN* convolutional neural network, *ML* machine learning.

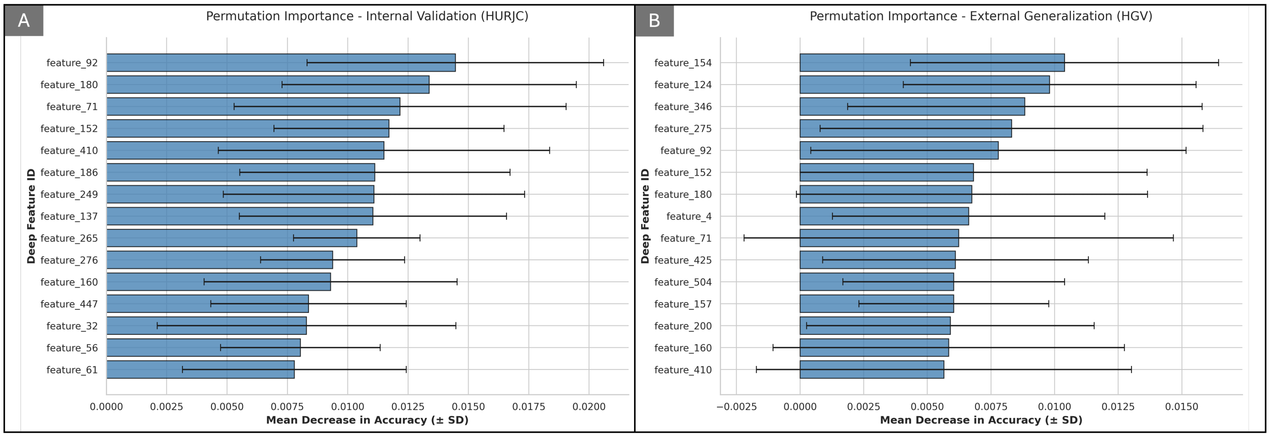

Figure S2. Permutation importance analysis for the hybrid CNN–SVM model on the (A) internal validation and (B) external generalization test sets.
